# Empirical studies on how ethical recommendations are translated into practice: a cross-section study on scope and study objectives

**DOI:** 10.1186/s12910-022-00873-x

**Published:** 2023-01-11

**Authors:** Johannes Schwietering, Holger Langhof, Daniel Strech

**Affiliations:** QUEST Center for Responsible Research, Berlin Institute of Health (BIH) at Charité – Universitätsmedizin Berlin, 10178 Berlin, Germany

**Keywords:** Empirical bioethics, Evaluation, Translational ethics

## Abstract

**Background:**

Empirical research can become relevant for bioethics in at least two ways. First, by informing the development or refinement of ethical recommendations. Second, by evaluating how ethical recommendations are translated into practice. This study aims to investigate the scope and objectives of empirical studies evaluating how ethical recommendations are translated into practice.

**Methods:**

A sample of the latest 400 publications from four bioethics journals was created and screened. All publications were included if they met one of the following three criteria: (1) evaluative empirical research, (2) non-evaluative empirical research and (3) borderline cases. For all publications categorized as evaluative empirical research we analyzed which objects (norms and recommendations) had been evaluated.

**Results:**

234 studies were included of which 54% (*n* = 126) were categorized as non-evaluative empirical studies, 36% (*n* = 84) as evaluative empirical studies, and 10% (*n* = 24) as borderline cases. The object of evaluation were aspirational norms in 5 of the 84 included evaluative empirical studies, more specific norms in 14 (16%) studies and concrete best practices in 65 (77%) studies. The specific best practices can be grouped under five broader categories: ethical procedures, ethical institutions, clinical or research practices, educational programs, and legal regulations.

**Conclusions:**

This mapping study shows that empirical evaluative studies can be found at all stages in the translational process from theory to best practices. Our study suggests two intertwined dimensions for structuring the field of evaluative/translational empirical studies in bioethics: First, three broader categories of evaluation objects and second five categories for types of best practices.

*Trial registration*: The methodology used was described in a study protocol that was registered publicly on the Open Science Framework (https://osf.io/r6h4y/).

**Supplementary Information:**

The online version contains supplementary material available at 10.1186/s12910-022-00873-x.

## Background

For a long time, bioethical research has been primarily concerned with the theoretical reflection of normative questions. This paradigm is based on a sharp separation of normative from descriptive ethics, which are fundamentally different in their epistemological interest as well as their employed methods. In the past two decades however, this separation has been increasingly overcome and a growing branch of empirical research in bioethics emerged. This change has been described as the empirical turn in bioethics [1]. Multiple authors presented evidence for the increase of empirical studies investigating ethical topics [2–4]. The most recent one showed that in a sample of nine bioethical journals, 18% (*n* = 1007) of the original papers collected and analyzed empirical data [3].

Empirical research can contribute to bioethics in several ways and several categorizations for these ways have been suggested, including studies on beliefs, perspectives, (new) issues, facts relevant to normative arguments, likely consequences or effectiveness [5–7].

**Fig. 1:**
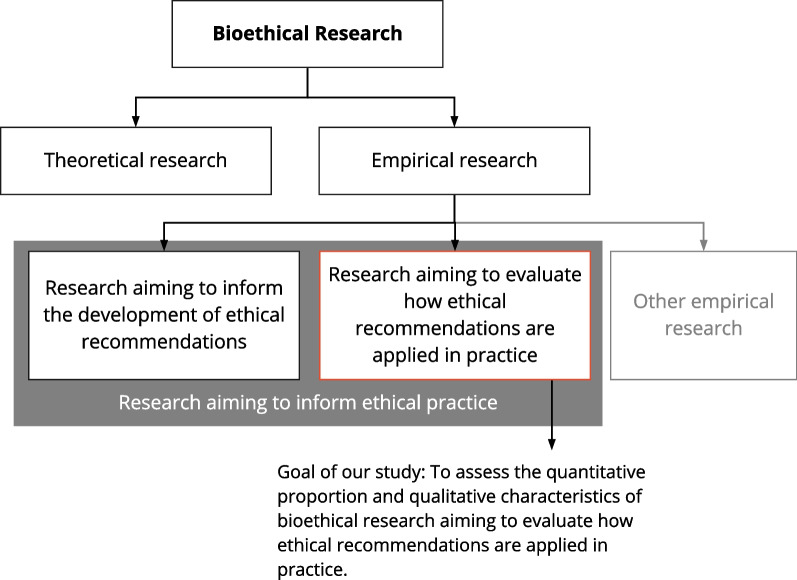
Two types of empirical research aiming to inform ethical practice

All these different ways can become relevant for bioethics in at least two ways (see Fig. 1). First, empirical research aims to inform the development or refinement of ethical recommendations. It can do so by informing about beliefs, perspectives, facts relevant to normative arguments, likely consequences or new ethical concerns [5–7]. For example, *attitudes research* such as surveys with different stakeholder groups might reveal important viewpoints on biobanking issues that should be acknowledged in developing practice oriented guidance [8]. Another example is the assessment of clinician’s experiences and views about ethical issues encountered in clinical practice to develop a clinical ethics support system [9].

A second complementary set of empirical studies aims to evaluate how effectively, efficiently, or valid the ethical recommendations are translated into practice [5–7]. For example, studies assessing whether and how clinical trials are prospectively registered and how they report their results clarifies the implementation of two ethical recommendations (principles 35 and 36) included in the Declaration of Helsinki [10–12]. Likewise, an empirical study assessing the understanding of informed consent materials provides empirical information that helps to understand whether the ethical recommendations of informed consent are implemented in a valid way [13–15]. More examples of this set of empirical studies are presented in Table 1 of the protocol.

**Table 1:** Definitions

Term	Definition
Evaluative empirical research	Empirical research evaluating how ethical recommendations are applied in practice. The publication was assigned to this category if it included a collection of either qualitative and/or quantitative data to evaluate the effectiveness (intended and unintended effects), efficiency or validity of the implementation of an ethical recommendation This may include studies that • Evaluate the extent to which an ethical recommendation is implemented in the current practice (e.g. status quo studies on how often results of completed clinical studies are published or to what extent informed consent forms follow the FDA recommendations on presenting risks and benefits) • Evaluate obstacles and opportunities that are associated with the implementation of the ethical recommendation (e.g. interview research with sponsors and principal investigators on experienced challenges and opportunities for implementing post-trial-access to drugs used in clinical trials) • Evaluate whether the ethical recommendation is implemented in a way that reflects the validity requirements of the respective recommendation. (e.g. a study investigating whether the concrete informed consent procedures of a specific study or in a specific context allow voluntary and informed consent decisions)
Non-evaluative empirical research	The publication was assigned to this category only if it includes a collection of either qualitative and/or quantitative data that is not used to assess the worth or merit of the implementation of an ethical recommendation This may include studies that • Assess the attitudes of stakeholders towards a medical procedure (e.g. interview research to assess the attitudes of lay people in Germany towards oocyt donation) • Aim to understand the expectations of stakeholders of medical procedures (e.g. interview animal researchers about their viewpoints on whether and how future animal studies should be registered or not)
Borderline cases	We expect that our categories are not absolutely distinct and there will be articles that cannot be clearly categorized. These borderline cases are valuable to further refine our concept This may include studies that • Provide a conceptual (armchair) evaluation of the effectiveness, efficiency or validity of ethical recommendations • Develop tools to evaluate the implementation of ethical recommendations (e.g. development of a scale to assess the understanding in an informed consent procedure)

In many areas that “scientifically” develop recommendations on how to act in certain situations (e.g. medical treatment recommendations) the recommendations are consecutively evaluated for how effectively and efficiently they can be and are implemented [16]. In the field of bioethics, however, multiple authors criticized a neglect of empirical research that evaluates the consequences of normative recommendations [6, 17, 18]. A recent review of practice evaluation of biobank ethics and governance conducted by our working group showed the need for more practice evaluation in the area of normative biobank governance [19].

The field of empirical studies evaluating how ethical recommendations are translated into practice has not been systematically investigated yet. To inform the methodological discourse of this field the aim of this cross-sectional study is a threefold mapping: First, to understand the current scope of this field we aim to map the quantitative proportions of evaluative empirical research published in leading bioethics journals (Journal of Medical Ethics, Nursing Ethics, AJOB Empirical Bioethics and the BMC Medical Ethics). Second, to understand the scope of overarching research objectives we want to map how often the evaluation object of empirical evaluative studies reflects either broad (aspirational) norms, specific norms, or best practices; a typology suggested by Sisk and colleagues [24]. Third, to understand the qualitative spectrum of the more specific research objectives of this field we aim to inductively map the specific objects of evaluation (the types of ethical recommendations).

## Methods

The methodology used was described in a study protocol that was registered publicly at the Open Science Framework (https://osf.io/r6h4y/).

### Sample size

The sample size was determined with the goal to reach thematic saturation for the assessment of the more specific study objectives. Former reviews on ethical issues and policies showed that the analysis of approximately hundred papers provides a qualitatively rich account of information that allows to reach thematic saturation for major categories [20]. However, this can only be estimated a priori as it is contingent on the categorization process.

### Search

To identify the quantitative proportion and methodological characteristics of evaluative empirical bioethical research in contrast to non-evaluative empirical research (see Fig. 1) a set of peer reviewed bioethics journals was used as a data source. We included the Journal of Medical Ethics and Nursing Ethics because a recent review by Wangmo et al. had shown that they publish the highest proportion of empirical research of all included journals [3]. Additionally, we included AJOB Empirical Bioethics and BMC Medical Ethics which were not part of the review by Wangmo et al. but also address empirical research in the fields of bioethics. To identify the latest 400 publications in these four journals (100 articles per journal) we used the journal categories from Pubmed by searching for the name of the journal and adding the term [Journal]. All hits were sorted by date and the first 100 (for each search) were downloaded as an XML file including the title and abstract.

### Eligibility screening

All downloaded studies were screened for exclusion based on title and abstract. The screening process was performed independently in a blinded standardized manner by 2 reviewers (JS and HL) and disagreements between reviewers were resolved by consensus. Interrater agreement was measured on a random subsample of *n* = 50 using Cohen’s kappa with sufficient reliability defined as *κ* > 0.8 (i.e. “very high” agreement) [21, 22]. To screen the title and abstract we used the open-source software Rayyan [23]. We excluded all publications that reported (1) purely theoretical studies, (2) literature reviews, (3) case studies, (4) no original researches, (5) duplicates. All other studies were included in the following step of the analysis.

### Categorization of included publication

All included publications were categorized in three categories: (1) evaluative empirical research, (2) non-evaluative empirical research and (3) borderline cases according to our predefined definitions (Table 1). The categorization was first conducted on the basis of the abstract and title of the publication. For all publications categorized as “evaluative empirical research” or “borderline cases” the full-text was retrieved and the categorization process was repeated based on the complete manuscript.

### Data extraction

For all publications categorized as evaluative empirical research we analyzed which objects (norms and recommendations) had been evaluated. Building on a publication from Sisk and colleagues we deductively grouped the objects under three categories [24]. The authors argue for an “implementation mindset” that ethicists should adopt when translating ethical norms from abstract normative claims to concrete changes in practice. The framework they introduce is composed of four sequential processes (i.e. (1) Normative Ethics, (2) Applied Ethics, (3) Intervention and (4) Dissemination Policy). The respective results from these sequential processes are then categorized in three levels: (1) Aspirational Norms, (2) Specific Norms, and (3) Best Practice. We chose this framework because it offers a compelling and clear structure for the translational process of ethical norms. We further analyzed which evaluative approaches have been used. All information were extracted as defined in the protocol (https://osf.io/r6h4y/).

The quality of the included studies was not assessed as this is not relevant for answering our research questions about scope and objectives.

### Analysis and statistics

The extracted data was analyzed by creating a data-driven coding frame as part of a qualitative content analysis [25]. For the qualitative content analysis the software MaxQDA (2020) was used [26]. To calculate summary measures (e.g. number of evaluative and non-evaluative studies) we used Microsoft® Excel for Mac version 16.61 (22,050,700).

## Result

### Search, inclusion and data extraction

**Fig. 2:**
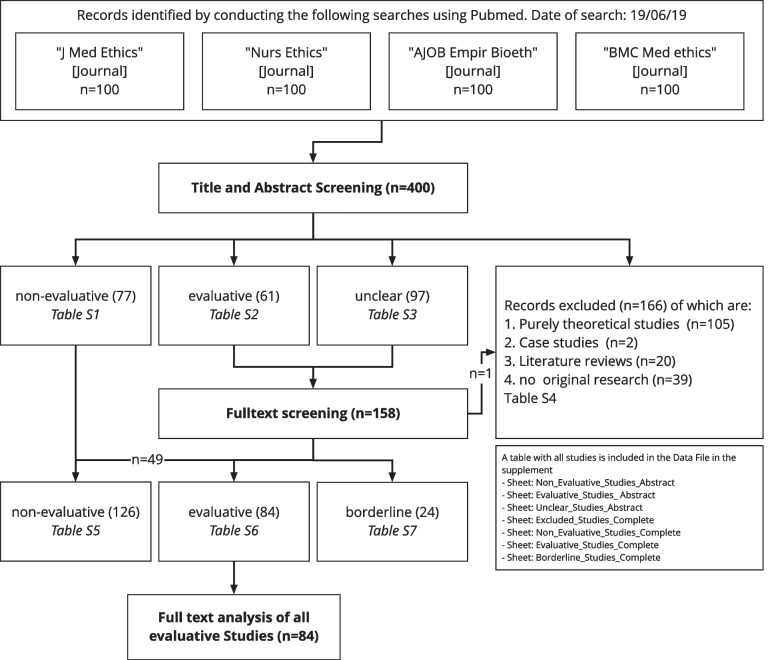
PRISMA Diagram of search and inclusion process

As a basis for our analysis, we identified 400 publications from four bioethics journals. The screening process led to the exclusion of 166 publications, most of which were purely theoretical studies (*n* = 105, 26%) (see PRISMA diagram [27], Fig. 2). The screening on title and abstract level showed high interrater reliability with Cohen’s Kappa of 0.82. Publication dates ranged from 2014 to 2019 with the majority of studies published 2019 (*n* = 254) or 2018 (*n* = 91).

### Evaluative and non-evaluative studies

**Fig. 3:**
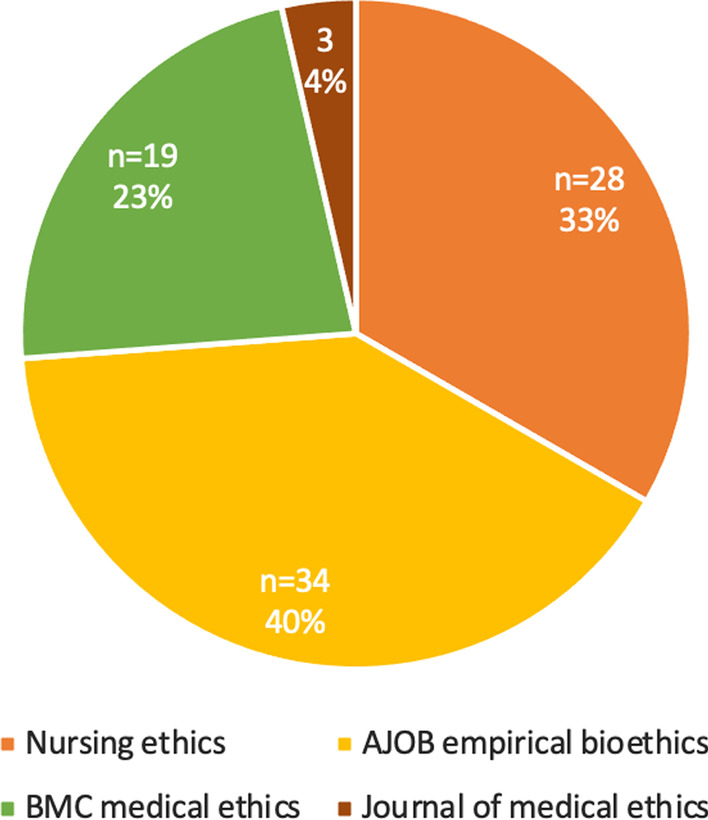
Number and percentage of evaluative studies per included journal

The categorization process of the 234 included empirical studies resulted in 54% (*n* = 126) being categorized as non-evaluative empirical studies, 36% (*n* = 84) as evaluative empirical studies, and 10% (*n* = 24) as borderline cases (Fig. 2). The 84 studies categorized as evaluative were included in the further data extraction process. Figure 3 shows the proportions and number of evaluative studies per included journal.

### Evaluation objects

**Fig. 4:**
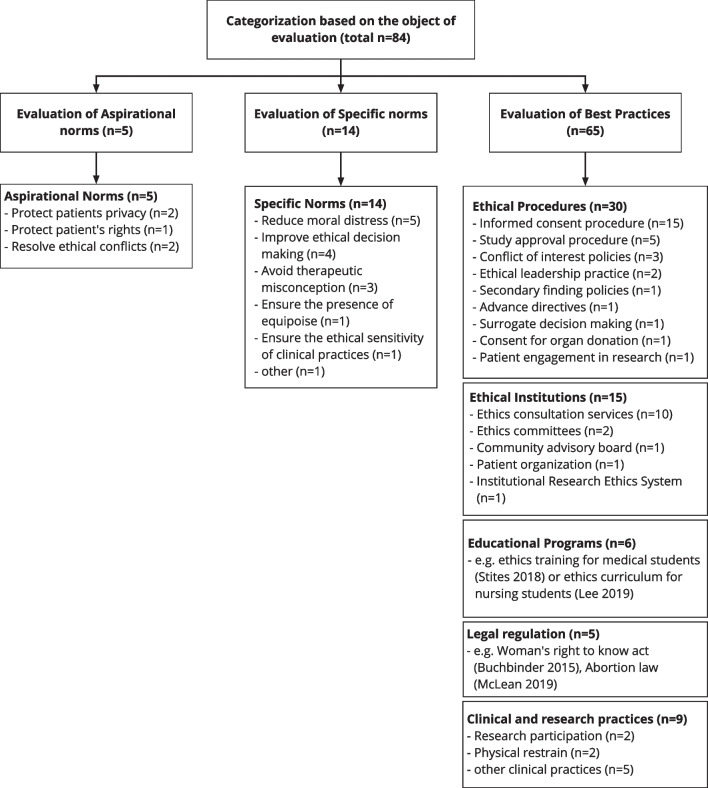
Categorization of evaluative empirical studies in bioethics

As described in the Methods section we grouped the evaluation objects from the evaluative empirical studies under the three categories: aspirational norms, specific norms, best practices. Additional file 1: Table S1 explains these categories further and gives examples from our sample. Figure 4 presents the topics studied for these three categories.

In five (6%) of all 84 evaluative empirical studies the evaluation object reflected very broadly formulated “aspirational norms” such as “protect patient rights” or “resolve ethical conflicts”. In 14 studies (16%) more “specific norms” were evaluated such as “reduce moral distress”, “improve ethical decision making”, or “avoid therapeutic misconception”. The majority of 65 studies (77%) evaluated concrete “best practices”.

We clustered the 65 studies evaluating best practices under five subgroups. A) “Ethical procedures” such as informed consent or study approval procedures were evaluated in 30 studies (36%), B) “ethical institutions” such as ethics consultation services or ethics committees were evaluated in 15 studies (18%), “clinical and research practices” were evaluated in 9 studies (11%), “educational programs” evaluated in 6 studies (7%), and “legal regulation” evaluated in 5 studies (6%).

Figure 4 presents more detailed information on the categorization of evaluative empirical studies. Additional file 2: Table S2 presents the complete categorization of the evaluative empirical studies (https://doi.org/10.17605/OSF.IO/R6H4).

## Discussion

This cross-sectional study aimed to map and categorize empirical studies published in bioethics journals that evaluate how ethical recommendations (practice-oriented theories) are translated into concrete decision making (practice). We do not know of any other study with a similar approach. We found that a substantial proportion (35%, *n* = 84) of all empirical studies included in our sample from four bioethics journals have such an evaluative objective.

This finding indicates that bioethics already has a subfield or a domain that studies the practice translation of ethical recommendations. Such as subfield would be in line with similar subfields in biomedicine or psychology where the evaluation of how effectively, efficiently or valid certain practice-oriented (treatment or prevention) recommendations are translated or implemented into practice is a field in its own. Translational research, implementation research, or health services research are prominent examples of such fields that have their own methods and terminologies. But in contrast to these subfields in medicine or psychology the evaluative studies on bioethical recommendations so far are not perceived as a field in itself. The bioethics literature so far primarily discussed the increased occurrence of empirical studies in general [2, 3], the (more or less legitimate) role of empirical studies in ethical reasoning [28] and the theory of evaluating the ethical practice [29, 30].

Our study suggests two intertwined dimensions for structuring the field of evaluative/translational empirical studies in bioethics. First, we chose a deductive approach according to distinguish three broader categories of evaluation objects [24]. Our study found that the majority of evaluative studies address the third category “best practices” but we also found studies that aimed to evaluate (broad) “aspirational norms” or “specific norms” without addressing a concrete best practice. Second, based on the 65 studies evaluating best practices we inductively developed five categories for types of best practices: c1) ethical procedures, c2) ethical institutions, c3) clinical or research practices, c4) educational programs, and c5) legal regulations.

Our study has limitations due to its explorative character and therefore both suggested dimensions for structuring the field of evaluative/translational bioethics need validation and might need refinement. For example, we used a broad definition of evaluative empirical research (see Table 1) to be sensitive in our analysis of this type of bioethical research. While we reached thematic saturation for these five categories for evaluation objects of best practices we need to stress that these categories were developed out of a sample of studies that were all published in only four bioethics journals. In these types of journals the most evaluated best practices were “informed consent”, “ethics consultation services”, and “study approval procedures”. These patterns might look different for empirical evaluative studies on ethics topics published in general medicine or specialty journals. Further studies including general medicine or specialty journals might help to validate or refine the five categories for types of best practices.

The results show a low proportion of evaluative empirical studies in the Journal of Medical Ethics compared to the three other included journals. Further research is needed to assess a connection between the amount of evaluative research and the type of journal. It was out of the scope of this explorative study to analyze the types of results that the empirical evaluative studies reported. Further research is needed to investigate to what extent evaluative studies actually help to improve the translation of ethical recommendations into practice. As introduced earlier, from a conceptual viewpoint the results of evaluation studies might address the effectiveness, efficiency or validity of the practice translations. Our study, however, did not empirically validate this conceptual distinction.

Sisk and colleagues call for ethicists to adopt an “implementation mindset” when formulating norms, and collaborate with others who have the expertise needed to implement policies and practices [24]. Evaluative empirical studies can provide the information needed to successfully translate ethical recommendations into practice and our study shows that these studies can be found at all stages in the translational process. We hope our mapping study facilitates discussions on how to further develop and assure quality of the emerging field of empirical studies on the practice translation of ethical recommendations.

## Supplementary Information

**Additional file 1: Table S1:** Categories based on value object of the evaluation.

**Additional file 2: Table S2:** Data extraction - Aspirational Norms.

## Data Availability

We will share our raw data and have therefore uploaded everything to our publicly accessible OSF project: https://osf.io/r6h4y/. The data is licensed under CC BY-NC 4.0. No additional source data is required.

## References

[1] Borry P, Schotsmans P, Dierickx K (2005). The birth of the empirical turn in bioethics. Bioethics.

[2] Borry P, Schotsmans P, Dierickx K (2006). Empirical research in bioethical journals. A quantitative analysis. J Med Ethics.

[3] Wangmo T, Hauri S, Gennet E, Anane-Sarpong E, Provoost V, Elger BS (2018). An update on the “empirical turn” in bioethics: analysis of empirical research in nine bioethics journals. BMC Med Ethics.

[4] Hurst S (2010). What ‘empirical turn in bioethics’?. Bioethics.

[5] Hope T (1999). Empirical medical ethics. J Med Ethics.

[6] Kon AA (2009). The role of empirical research in bioethics. Am J Bioeth.

[7] Sugarman J, Sulmasy DP, editors. Methods in medical ethics. 2nd ed. Washington, D.C: Georgetown University Press; 2010; 353.

[8] Abdelhafiz AS, Sultan EA, Ziady HH, Ahmed E, Khairy WA, Sayed DM (2019). What Egyptians think knowledge, attitude, and opinions of egyptian patients towards biobanking issues. BMC Med Ethics.

[9] Fuscaldo G, Cadwell M, Wallis K, Fry L, Rogers M (2019). Developing clinical ethics support for an Australian health service: a survey of clinician’s experiences and views. AJOB Empir Bioeth.

[10] Riedel N, Wieschowski S, Bruckner T, Holst MR, Kahrass H, Nury E, et al. Results dissemination from completed clinical trials conducted at German university medical centers remained delayed and incomplete. The 2014 –2017 cohort. J Clin Epidemiol. 2022;144:1–7. 10.1016/j.jclinepi.2021.12.012 34906673

[11] World medical association declaration of Helsinki: ethical principles for medical research involving human subjects. JAMA. 2013;310(20):2191. 10.1001/jama.2013.281053 24141714

[12] Chen R, Desai NR, Ross JS, Zhang W, Chau KH, Wayda B (2016). Publication and reporting of clinical trial results: cross sectional analysis across academic medical centers. BMJ.

[13] Sommers R, Van Staden C, Steffens F (2017). Views of clinical trial participants on the readability and their understanding of informed consent documents. AJOB Empir Bioeth.

[14] Pietrzykowski T, Smilowska K (2021). The reality of informed consent: empirical studies on patient comprehension—systematic review. Trials.

[15] Kahrass H, Bossert S, Schürmann C, Strech D (2021). Details of risk–benefit communication in informed consent documents for phase I/II trials. Clin Trials.

[16] Woolf SH. The meaning of translational research and why it matters. JAMA [Internet]. 2008; 10.1001/jama.2007.26 10.1001/jama.2007.26 18182604

[17] De Vries R (2004). How can we help? From “sociology in” to “sociology of” bioethics. J Law Med Ethics.

[18] Leget C, Borry P, De Vries R (2009). “Nobody tosses a dwarf!” the relation between the empirical and the normative reexamined. Bioethics.

[19] Langhof H, Schwietering J, Strech D (2019). Practice evaluation of biobank ethics and governance: current needs and future perspectives. J Med Genet.

[20] Klingler C, Silva DS, Schuermann C, Reis AA, Saxena A, Strech D (2017). Ethical issues in public health surveillance: a systematic qualitative review. BMC Public Health.

[21] Brennan RL, Prediger DJ (1981). Coefficient Kappa: some uses, misuses, and alternatives. Educ Psychol Meas.

[22] Landis JR, Koch GG (1977). The measurement of observer agreement for categorical data. Biometrics.

[23] Ouzzani M, Hammady H, Fedorowicz Z, Elmagarmid A (2016). Rayyan—a web and mobile app for systematic reviews. Syst Rev.

[24] Sisk BA, Mozersky J, Antes AL, DuBois JM (2020). The “ought-is” problem: an implementation science framework for translating ethical norms into practice. Am J Bioeth.

[25] Schreier M. Qualitative content analysis in practice. Los Angeles: SAGE; 2012. 272.

[26] VERBI Software. (2019). MAXQDA 2020 [computer software]. Berlin, Germany: VERBI Software. Available from maxqda.com.

[27] PRISMA-P Group, Moher D, Shamseer L, Clarke M, Ghersi D, Liberati A, et al. Preferred reporting items for systematic review and meta-analysis protocols (PRISMA-P) 2015 statement. Syst Rev [Internet]. 2015 Dec [cited 2019 Oct 9];4(1). Available from: https://systematicreviewsjournal.biomedcentral.com/articles/10.1186/2046-4053-4-1 10.1186/2046-4053-4-1 PMC4320440 25554246

[28] Ives J, Dunn M, Molewijk B, Schildmann J, Bærøe K, Frith L (2018). Standards of practice in empirical bioethics research: towards a consensus. BMC Med Ethics.

[29] Cribb A (2010). Translational ethics? The theory-practice gap in medical ethics. J Med Ethics.

[30] Bærøe K (2014). Translational ethics: an analytical framework of translational movements between theory and practice and a sketch of a comprehensive approach. BMC Med Ethics.

