# Supplementary material for: Empirical studies on how ethical recommendations are translated into practice: a cross-section study on scope and study objectives
Source: BMC Med Ethics. 2023 Jan 11;24:2. doi: 10.1186/s12910-022-00873-x (PMC9835353; doi:10.1186/s12910-022-00873-x)
Supplement: Supplementary file 1 — Additional file 1: Table S1: Categories based on value object of the evaluation. [file 12910_2022_873_MOESM1_ESM.docx]

**Supplement Table 1: Categories based on value object of the evaluation**

| **Normative Stage** | **Explanation from Sisk (2020)** | **Examples from our sample** |
| --- | --- | --- |
| Aspirational Norm | Deliberations in normative ethics may result in what we call aspirational norms, which are broad claims that are easily agreed upon but difficult (or impossible) to implement. An aspirational normative claim might be that “No one in the world should die of hunger.” This claim would garner broad agreement, but it does not specify any particular actions. Such aspirational norms are valuable insofar as they serve as a “true North” to guide subsequent development of specific norms. Once an aspirational norm is developed, deliberations in applied ethics can generate specific norms. | Eklöf, Hupli and Leino-Klpi: Factors related to privacy of Somali refugees in health care (1)   - Aim: to describe the factors related to the realisation of privacy of Somali refugees in health care by describing the factors related to the patient, healthcare professional and interpreter.   Abdalla et al.: Operationalization of patients’ rights in Sudan: Quantifying nurses’ knowledge (2)   - Aim: to assess the level of knowledge about patients’ rights among the nursing staff at Friendship Teaching Hospital in Sudan. |
| Specific Norm | These specific norms provide directed guidance about the types of actions that ought to be enacted. Consider the following normative claim: “Every physician in the United States should question their patients about food insecurity and provide information packets about local resources to protect them from hunger.” This claim is specific and more feasible to be implemented. Although this specific normative claim will not fully rid the world of hunger, it provides an incremental step toward this broader aspirational norm. | Lazzari et al.: Moral distress in correctional nurses: A national survey (3)   - Aim: To investigate the level of moral distress of nurses working in the Italian correctional setting, by completing the validation process of the Moral Distress Scale for Correctional Nurses.   Torabi et al.: Barriers to ethical decision-making for pre-hospital care professionals (4)   - Aim: The purpose of this study was to identify barriers of ethical decision-making in Iranian Emergency Medical Service personnel. |
| Best Practice | After a specific norm is formulated, investigators can develop interventions to enact this norm. These interventions will provide measurable outcomes to determine whether the intervention has succeeded or failed to enact the specific norm. As data accumulate from studies of interventions, investigators and ethicists can identify best practices. Once these best practices are identified, they should be disseminated broadly, occasionally with the backing of policy change. | Master et al.: Conflicts of interest policies for authors, peer reviewers, and editors of bioethics journals (5)   - Aim: To evaluate the availability and comprehensiveness of conflict of interest policies   Somers, Van Staden and Steffens: Views of clinical trial participants on the readability and their understanding of informed consent documents (6)   - Aim: To evaluate the readability and subjective understanding of deidentified standard informed consent document. |

1. Eklöf N, Hupli M, Leino-Kilpi H. Factors related to privacy of Somali refugees in health care. Nurs Ethics. 2020 Mar;27(2):514–26.

2. Abdalla SM, Mahgoub EA, Abdelgadir J, Elhassan N, Omer Z. Operationalization of patients’ rights in Sudan: Quantifying nurses’ knowledge. Nurs Ethics. 2018 Oct;969733018787224.

3. Lazzari T, Terzoni S, Destrebecq A, Meani L, Bonetti L, Ferrara P. Moral distress in correctional nurses: A national survey. Nurs Ethics. 2019 Apr;969733019834976.

4. Torabi M, Borhani F, Abbaszadeh A, Atashzadeh-Shoorideh F. Barriers to ethical decision-making for pre-hospital care professionals. Nurs Ethics. 2019 Jun;969733019848044.

5. Master Z, Werner K, Smith E, Resnik DB, Williams-Jones B. Conflicts of interest policies for authors, peer reviewers, and editors of bioethics journals. AJOB Empir Bioeth. 2018 Jul;9(3):194–205.

6. Somers R, Van Staden C, Steffens F. Views of clinical trial participants on the readability and their understanding of informed consent documents. AJOB Empir Bioeth. 2017 Oct;8(4):277–84.
